# Case Report: Nursing strategies for colon cancer surgery in third-trimester pregnancy

**DOI:** 10.3389/fonc.2025.1683647

**Published:** 2025-11-28

**Authors:** Shiping Zeng, Jiayou Chen, Yan Luo, Dan Song

**Affiliations:** General Medicine Department, Affiliated Hospital of Chengdu University, Chengdu University, Chengdu, China

**Keywords:** case report, main nursing diagnosis, colorectal cancer, pregnancy, intervention

## Abstract

**Background:**

Colorectal cancer during pregnancy is rare and poses significant challenges for maternal and fetal care. Postoperative nursing interventions are essential to optimize recovery and prevent complications.

**Case presentation:**

A 33-year-old primigravida (G1P0) at 29^5^ weeks’ gestation presented with abdominal distension, lower back pain, anorexia, and fatigue. Imaging and laboratory tests revealed right ascending colon wall thickening, hepatic lesions, fecal occult blood positivity, elevated AFP (162.4 IU/mL) and CA125 (64.5 U/mL), and severe anemia. She underwent cesarean section with right hemicolectomy, D3 lymph node dissection, and partial hepatectomy. Postoperative nursing care included pain management via patient-controlled analgesia, parenteral and oral nutrition, fluid and electrolyte monitoring, drainage tube care, early mobilization, fever surveillance, deep vein thrombosis prophylaxis, psychological support, and maternal-neonatal separation management.

**Outcome:**

The patient recovered progressively without subjective complaints. No incision infection, vaginal fluid leakage, or mastitis occurred. The surgical incision healed with Grade A outcome, and she was discharged in stable condition.

**Conclusion:**

This case demonstrates the effectiveness of integrated, evidence-based postoperative nursing strategies in managing colorectal cancer during late pregnancy, providing guidance for similar complex cases.

## 1 Introduction

Colon cancer, ranking as the third most prevalent malignancy globally, accounts for over 1 million cancer-related deaths annually (1). In China, it currently stands as the fourth leading cause of cancer incidence and mortality, following gastric, esophageal, and lung cancers. In recent years, with changes in dietary structure, the incidence has shown an increasing trend year by year (2). Although rare, colon cancer during pregnancy presents unique diagnostic and therapeutic challenges due to overlapping symptoms with normal gestation and concerns about fetal safety. Its incidence is estimated at approximately 1 in 13,000 pregnancies, often leading to delayed diagnosis and poorer maternal outcomes (3, 4). The clinical management of colon cancer complicated by third-trimester pregnancy presents exceptional challenges, as the combined procedures of cesarean section and radical colectomy may impose significant physical and psychological stress, increasing risks of postoperative complications.

To address these challenges, our institution adopted a multidisciplinary collaborative model for a representative case in July 2017. A patient diagnosed with right-sided colon cancer during late pregnancy underwent coordinated surgical intervention by obstetrics and general surgery teams, followed by intensive postoperative care across ICU, obstetrics, and surgical departments. Through implementation of evidence-based comprehensive nursing protocols, the patient achieved successful recovery with mitigated complications. This report details the integrated clinical strategy and outcomes, providing actionable insights for managing similar complex cases.

## 2 Case presentation

### 2.1 Patient information

A 33-year-old primigravida (Gravida 1, Para 0, Abortus 0, 29^5^ weeks’ gestation) was admitted to the obstetrics department on July 18, 2017, presenting with a 2-month history of lower back pain and abdominal distension, as well as 1 week of anorexia and fatigue. Initial clinical assessment revealed moderate anemia (hemoglobin 73 g/L), a 29^5^-week intrauterine pregnancy with breech presentation, and undifferentiated abdominal pain with suspected intestinal pathology. The chronology of clinical events, nursing interventions, and patient outcomes is summarized in **Table 1**, providing a clear overview of the multidisciplinary care and postoperative recovery.

**Table 1:** Chronology of clinical events, nursing interventions, and outcomes in a patient with colorectal cancer during late pregnancy.

Date	Event/examination	Nursing/clinical interventions	Outcome/changes
July 18, 2017	Admission (29^5^ weeks gestation)	- Initial assessment: labs, imaging, tumor markers - Fetal heart rate and structural monitoring	Severe anemia (Hb 73 g/L), right ascending colon wall thickening, hepatic lesions, fetal bilateral pleural effusion detected
July 20 & 26, 2017	Preoperative anemia correction	- Transfusion of 4 units A Rh(D)+ packed RBCs - Monitoring BP, HR, and fetal heart rate	Hb improved; fetal monitoring stable
July 27, 2017	Combined surgery (cesarean section + right hemicolectomy + D3 lymph node dissection + partial hepatectomy)	- ICU monitoring: central venous pressure - PCA pain management - Antibiotics and PPI therapy - Fluid and electrolyte management	Surgery completed successfully; intraoperative frozen pathology confirmed poorly differentiated adenocarcinoma (pT3N1bM1)
July 28, 2017	Transfer to general surgery ward	- Initiation of parenteral nutrition (PN 2,000 kcal/day) - Gradual oral intake (clear liquids → soft diet) - Drainage tube care, incision observation, positioning	Gastrointestinal function gradually recovered; drainage smooth; incision intact
July 28 – August 13, 2017	Postoperative recovery	- Pain management and PCA education - Fluid and electrolyte monitoring - Early mobilization and DVT prophylaxis - Fever monitoring and infection prevention - Psychological support and maternal–neonatal separation management - Complication prevention (mastitis, surgical site infection, hemorrhage)	Patient reported no discomfort; incision healed well; no infection or mastitis; psychological status stable
August 14, 2017	Initiation of adjuvant chemotherapy	- Chemotherapy administration (Calcium folinate 200 mg/m² + 5-FU 500 mg/m²) - Patient/family education on side effects, adherence, and oral care	Tumor markers decreased significantly (AFP 58.7 IU/mL, CA125 32.1 U/mL)
Discharge	Recovery and follow-up	- Activities of daily living assessment - Health education and chemotherapy adherence guidance	Surgical incision Grade A; independent in daily activities; 5-year follow-up showed no recurrence and good overall health

- Abdominal Ultrasound: The liver was of normal size with smooth capsule and homogeneous parenchymal echogenicity. A slightly hyperechoic lesion measuring approximately 1.4 × 1.3 cm with clear borders and regular shape was noted in the right posterior superior segment, while another hypoechoic lesion measuring about 2.6 × 1.7 cm with irregular shape and heterogeneous internal echo was found in the right posterior inferior segment; neither showed significant blood flow on Doppler imaging. The gallbladder, pancreas, spleen, and kidneys were unremarkable. No rectal bleeding was reported.
- Obstetric Ultrasound: Single intrauterine pregnancy in breech presentation. Fetal biometry showed biparietal diameter 7.4 cm and femur length 5.6 cm. Placenta located on the posterior wall with a thickness of 2.9 cm (Grade I maturity), maximum amniotic fluid depth 3.4 cm. Umbilical artery Doppler: Vp 47.8 cm/s, Vmin 15.9 cm/s, RI 0.67, S/D 3.0. Fetal heart rate 141 bpm. Fetal structural evaluation demonstrated normal cranial contour with midline symmetry, continuous spinal echo, visible four-chamber heart, and identifiable liver, stomach, kidneys, and bladder without renal pelvis dilation.
- MRI Findings: Right ascending colon wall thickening with hepatic lesions, raising concerns for malignant neoplasia or lymphoma, alongside fetal bilateral pleural effusion. Fetal imaging showed no structural abnormalities.
- Laboratory Investigations: Fecal occult blood positive via immunochemical testing, elevated tumor markers (AFP 162.4 IU/mL, CA125 64.5 U/mL), and severe anemia necessitating pre-admission transfusion of 8.5 units of packed red blood cells.
- Medical History: Appendectomy in 2007 and prior enteritis in May 2016. Preoperative stabilization involved two additional transfusions of A Rh(D)+ packed red blood cells (4 units total on July 20 and 26) alongside continuous fetal monitoring, which showed reassuring cardiotocographic patterns.

### 2.2 Surgical intervention

On July 27, 2017, a multidisciplinary surgical team performed combined cesarean delivery and oncological resection under spinal-epidural anesthesia. The procedure included a cesarean section, right hemicolectomy with D3 lymph node dissection, partial hepatectomy of segment VI, and intraoperative frozen section analysis confirming poorly differentiated adenocarcinoma (pathological staging: pT3N1bM1). Postoperatively, the patient was transferred to the ICU for hemodynamic stabilization, where management included central venous pressure monitoring (maintained at 8–12 cmH_2_O), patient-controlled analgesia with fentanyl, oxytocin infusion (20 IU/24 h) to promote uterine involution, empirical antimicrobial coverage using cefoperazone-sulbactam, proton pump inhibitor therapy for gastric protection, and fluid resuscitation to maintain electrolyte balance.

On July 28, the patient was transferred to the general surgery ward for continued recovery. Parenteral nutrition (2,000 kcal/day) was initiated, progressing to oral intake by postoperative day 4, advancing from clear liquids to a soft diet. Given the diagnosis of metastatic colorectal cancer, adjuvant chemotherapy with calcium folinate (200 mg/m²) and fluorouracil (500 mg/m²) was commenced on August 14 following confirmation of hemostasis and absence of vaginal bleeding. Serial tumor marker surveillance demonstrated a significant reduction (AFP 58.7 IU/mL, CA125 32.1 U/mL by postoperative day 14), supporting therapeutic efficacy.

Throughout hospitalization, coordinated care between obstetrics, surgical, and intensive care teams ensured comprehensive management of coexisting obstetric and oncological challenges. The treatment approach incorporated enhanced recovery principles and individualized psychosocial support to address the complexities of perinatal malignancy.

### 2.3 Perioperative nursing team

The perioperative care was provided by a dedicated team of 6 specialized nurses, including 2 obstetric nurses, 2 general surgical nurses, and 2 ICU nurses. The team operated under a coordinated schedule to provide continuous monitoring and timely intervention. Nurses were responsible for pain management, nutritional support, fluid and electrolyte balance, drainage tube care, mobilization, psychological support, and proactive prevention of complications, in collaboration with physicians and dietitians.

### 2.4 Methods of nursing and postoperative care

#### 2.4.1 Pain management

Effective pain management is fundamental to enhanced postoperative recovery. Adequate pain relief not only increases patient comfort and improves the quality of postoperative recovery but also reduces stress responses, promotes early mobilization, and facilitates the early resumption of oral intake (5). The responsible nurse accurately assesses the cause of the patient’s current pain and educates the patient that pain may result from postpartum uterine contractions, abdominal incisions, breast engorgement, and other factors, thereby alleviating anxiety and concerns. The patient is provided with a patient-controlled analgesia (PCA) pump, and the nurse explains its proper use to both the patient and family members (6). The responsible nurse collaborates with obstetric specialists to monitor postpartum recovery, including assessing uterine involution, measuring fundal height, and observing the color and volume of lochia.

A comprehensive multimodal analgesic regimen was implemented perioperatively. Preoperatively, intramuscular pethidine was administered as needed to relieve tumor-related abdominal pain and enhance surgical tolerance. Intraoperatively, standard anesthetic and analgesic techniques were used to maintain hemodynamic stability and minimize nociceptive input. Postoperatively, patient-controlled intravenous analgesia (PCIA) was initiated immediately after surgery. Analgesic management was further tailored to the care setting: in the ICU, butorphanol tartrate 10 mg in 40 mL normal saline was continuously infused via pump, whereas in the general surgery ward, tramadol, clonixin, and pethidine were administered as needed for breakthrough pain.

#### 2.4.2 Nutritional support

Patients with colorectal cancer require preoperative and postoperative fasting, which can impair gastrointestinal function, alter systemic metabolism, and lead to malnutrition in most cases. Therefore, evidence-based nutritional support is critical for postoperative recovery. Current clinical nutritional strategies primarily include parenteral nutrition (PN) and enteral nutrition (EN) (7). Due to postoperative fasting, hypoproteinemia, and nutritional imbalance, the patient receives intravenous infusions of Compound Amino Acid Injection (18AA-II), human albumin, and medium/long-chain fat emulsion (C6-24). While PN effectively meets postoperative nutritional needs, it may contribute to intestinal immune dysfunction and intolerance in some patients (7). Amino acids and fat emulsions are essential macronutrients; rapid infusion can trigger adverse reactions such as nausea, vomiting, and arrhythmias. PN delivery routes include peripheral veins and central venous access (e.g., central venous catheter [CVC], peripherally inserted central catheter [PICC], or implanted port [PORT]). The choice of route should balance clinical efficacy, cost-effectiveness, and complication prevention, tailored to the patient’s condition and institutional resources (8).

For this patient with good vascular integrity, peripheral venous access is selected. The nurse assesses vascular suitability, prioritizing large, straight, and elastic veins for cannulation. During infusion, the nurse closely monitors the insertion site for pain and observes for systemic reactions such as fever, palpitations, nausea, vomiting, or anaphylactic shock. To address hypoproteinemia, human albumin is administered sequentially after amino acids and fat emulsions. The nurse adjusts the infusion sequence appropriately, ensuring the use of a new infusion set for albumin following PN solutions.

#### 2.4.3 Dietary care

After postoperative gastrointestinal function recovery, the patient may resume oral intake, transitioning gradually from a clear liquid diet to a full liquid diet and eventually to a regular diet, following a small, frequent meal pattern with incremental portion adjustments. To optimize nutritional intake, the attending nurse collaborates with a dietitian to develop a personalized meal plan tailored to the patient’s clinical status and physiological needs. The diet emphasizes high-calorie, high-protein, and vitamin-rich low-residue foods. The patient is advised to consume smaller, more frequent meals to facilitate digestion. To prevent abdominal distension, constipation, or difficult bowel movements, the nurse educates the patient and family on avoiding gas-producing foods (e.g., onions, garlic, legumes, sweet potatoes) and excessive dietary fiber intake. The nurse monitors and documents the patient’s food intake, dietary habits, and bowel movements, assessing nutritional status and ensuring bowel regularity (9).

#### 2.4.4 Fever management

Postoperative stress responses may trigger recurrent fever, with temperatures peaking at 37.8 °C. To prevent infections, antibiotics are administered as prescribed. Surgical incision care is prioritized, including regular dressing changes to minimize infection risks. Perineal care is performed twice daily to maintain hygiene, monitor lochia characteristics (color, volume, and odor), and prevent puerperal or ascending urinary tract infections. For temperatures ranging from 37.5 °C to 38.0 °C, non-pharmacological interventions include ensuring proper room ventilation, applying physical cooling methods such as tepid sponging, changing sweat-soaked clothing, and maintaining clean, dry bedding to enhance comfort. Temperature monitoring is conducted every 30 minutes after intervention, then every 4 hours until normalization. If afebrile for three consecutive days, monitoring frequency is reduced to twice daily (bid), with all findings recorded accordingly (10).

#### 2.4.5 Fluid and electrolyte imbalance

Postpartum fluid loss, including excessive sweating, combined with postoperative fasting, increases the risk of dehydration and electrolyte imbalances, particularly sodium depletion. Fluid management includes calculating daily fluid requirements based on basal needs, ongoing losses, and existing deficits. The attending nurse closely monitors the 24-hour intake/output (I/O) balance and urine volume, tracking fluid status trends to promptly detect dehydration or electrolyte disturbances. Adjustments to intravenous or oral rehydration are made based on clinical assessments and laboratory findings.

#### 2.4.6 Drainage tube care

The patient is equipped with two postoperative plasma drainage tubes, requiring meticulous care to optimize surgical outcomes and facilitate recovery. Proper fixation of the tubes is emphasized, with repeated education provided to the patient and family regarding their importance and correct positioning. Maintaining drainage tube patency is a priority, achieved by routinely checking for obstructions or kinks. The nurse continuously monitors the drainage fluid characteristics, including color, consistency, and volume, to detect abnormalities such as infection or hemorrhage. The insertion site is kept clean and dry, with scheduled dressing changes to prevent infections. Drainage tubes are removed only after ultrasound reassessment confirms reduced output and normal fluid properties. Secure fixation and patient education help prevent accidental dislodgement (9).

#### 2.4.7 Mobilization

Postoperative management was conducted in accordance with the Enhanced Recovery After Surgery (ERAS) guidelines for colorectal surgery, following the recommendations of the ERAS Society (11, 12). Standard ERAS measures, including early ambulation, multimodal analgesia, early oral feeding, and prevention of postoperative ileus, were implemented to promote recovery and reduce complications. During bed rest, the patient is guided to perform limb exercises and frequent positional changes. Once medically stable, gradual ambulation is initiated to stimulate intestinal motility, reduce abdominal distension, and minimize risks of adhesions and deep vein thrombosis (DVT). Mobilization efforts are tailored to address potential barriers, including postoperative fatigue, preoperative physical deconditioning, catheter-related restrictions, and psychological hesitancy. Throughout mobilization, precautions are taken to protect surgical incisions and drainage tubes from tension or displacement, ensuring patient safety and comfort (13).

#### 2.4.8 Psychological support

The patient exhibited anxiety and emotional distress linked to preterm delivery, hormonal fluctuations, and cancer-related uncertainties. A multidisciplinary approach was adopted, with the medical team conducting regular consultations to align treatment plans and foster transparent communication with the patient and family. Psychological support was primarily provided by the nursing team through individualized interventions and empathetic communication. We acknowledge that support from non-specialized nurses or physicians may be insufficient, and hospitals should implement dedicated psycho-oncological services to address the complex emotional needs of cancer patients (14, 15). Comprehensive health education was provided to demystify the disease process, enhance treatment adherence, and rebuild confidence. Family members were trained in caregiving techniques and encouraged to offer emotional support, creating a holistic recovery environment.

#### 2.4.9 Maternal-neonatal separation

The preterm infant was transferred to the neonatal intensive care unit (NICU) at a maternal and child health hospital for specialized treatment. Maternal-neonatal separation created psychological challenges for the mother, including difficulty adapting to her postpartum role and anxiety about the infant’s prognosis. To address this, the nursing team implemented early identification of maternal emotional distress and provided empathetic communication to encourage expression of concerns. Maternal anxiety was assessed both before and after childbirth using the State-Trait Anxiety Inventory (STAI). Prepartum evaluation was conducted during hospitalization prior to delivery. Postpartum assessment was performed on day 3 after delivery, at which time the patient had already spent the majority of the postoperative period in the ICU. By the time she was transferred to the general ward, her condition was stable and gradual oral intake had been initiated. Overall, postoperative recovery following the major abdominal surgery was satisfactory. To address maternal emotional distress, the nursing team implemented early identification and empathetic communication to encourage expression of concerns. Family members were instructed to share daily photos or videos of the infant, fostering maternal-infant connection and helping to alleviate anxiety.

#### 2.4.10 Proactive management of postoperative complications

Late postpartum hemorrhage may occur due to uterine atony or other factors following cesarean section. Close monitoring of uterine contraction strength and lochia volume is essential, with timely pharmacological intervention (e.g., uterotonics) if necessary. Mastitis prevention is crucial, particularly in patients who cannot breastfeed due to postoperative fasting. Strategies include maintaining lactation patency and administering roasted barley tea after resuming oral intake to suppress milk production and prevent engorgement.

Surgical site infection (SSI) and anastomotic leak require vigilant monitoring of vital signs, incision characteristics (e.g., redness, swelling, severe pain), and drainage fluid properties (16). Prophylactic antibiotics, strict aseptic wound care, and regular dressing changes are implemented to prevent infection. Patients with poor nutritional status require close observation for sudden or worsening abdominal pain or cloudy drainage fluid, with enemas strictly avoided within 7–10 days postoperatively to minimize the risk of anastomotic leak.

Lower-extremity DVT is a concern due to prolonged bed rest, intraoperative factors, and anesthetic effects, all of which contribute to slowed venous circulation. Preventive measures include frequent limb assessments for temperature, skin color, and arterial pulse abnormalities. Patients are encouraged to perform ankle pump exercises, soak their feet in warm water twice daily, and ambulate as early as possible once their condition permits to reduce thrombosis risk.

Chemotherapy-induced gastrointestinal toxicity necessitates pre-treatment counseling on the importance of chemotherapy adherence and side effect management. Patients and families are educated on proper oral hygiene, including warm saline rinses before and after meals, to minimize mucosal irritation. Pharmacological interventions such as antiemetics are administered as prescribed to alleviate gastrointestinal symptoms.

### 2.5 Outcomes

Following over one month of comprehensive management, including anti-infective therapy, nutritional support, gastric acid suppression, complication prevention, and antitumor treatment, the patient’s condition improved significantly. She reported no discomfort and achieved independence in daily living, with a Barthel Index score reflecting self-sufficiency. No complications, such as pressure ulcers, wound infections (the incision site remained free of redness, exudate, or vaginal discharge), or mastitis secondary to milk stasis, were observed. The surgical incision healed optimally (Grade A), and she was discharged for continued recovery. Post-discharge, the patient adhered to a standardized chemotherapy regimen. Five-year follow-up confirmed sustained remission, with stable laboratory parameters and good overall health.

## 3 Discussion

Colorectal cancer is a common malignancy, and perioperative nursing quality plays a crucial role in postoperative outcomes. In this case, the patient underwent cesarean section combined with right hemicolectomy, partial hepatectomy, and intra-abdominal tumor management, highlighting the complexity of pregnancy-associated colorectal cancer. Pathology revealed a 13 × 9 × 2 cm moderately to poorly differentiated ulcerative adenocarcinoma with mucinous and signet-ring components, invading pericolonic fat with vascular tumor emboli and metastasis in 2 of 14 lymph nodes. Resection margins were negative except for focal distal mucosal polypoid proliferation. Immunohistochemistry showed AE1/AE3(+), CK20(+), CDX2(+), Ki-67 (~50%), Villin(+), CD34(+ in vascular emboli), D2-40(+ in lymphovascular emboli), and EGFR(+). The presence of mucinous and signet-ring features with lymphovascular invasion indicates aggressive tumor behavior and poor prognosis, underscoring the need for radical surgery, individualized adjuvant therapy, and close follow-up.

Effective perioperative pain management is pivotal for enhancing recovery, promoting gastrointestinal function, and ensuring patient comfort. Inadequately controlled pain can trigger stress responses, impair wound healing, and delay mobilization. In this case, a proactive, multimodal analgesic approach was employed. Preoperatively, intramuscular pethidine was administered for cancer-related abdominal discomfort. Intraoperatively, standard anesthetic techniques ensured hemodynamic stability while minimizing nociceptive input. Postoperatively, PCIA was maintained for 48 hours, supplemented by tailored analgesics in the ICU and general ward to manage breakthrough pain. This comprehensive strategy not only minimized opioid requirements but also facilitated early mobilization, accelerated gastrointestinal recovery, and improved overall postoperative outcomes. The case underscores that individualized, multimodal perioperative analgesia is a key component of enhanced recovery protocols, particularly for complex obstetric-oncologic patients.

The surgical incision, located 1 cm to the right of the lower abdominal midline, was a vertical incision approximately 12 cm in length. Within the first 48 hours, the incision exhibited mild erythema and swelling, accompanied by moderate pain (VAS 5-6). Standardized wound care and ongoing analgesia led to subsidence of local inflammation and a decrease in pain scores to VAS 2–3 by postoperative day 3, indicating effective pain control and satisfactory wound healing.

In addition to pharmacological interventions, integrated, evidence-based perioperative nursing—including prophylactic anti-inflammatory measures and nutritional support (PN + EN)—further optimized immune function, promoted recovery, and prevented complications such as impaired wound healing and intestinal barrier dysfunction.

Nutritional management was guided by comprehensive diagnostic assessment and clinical monitoring throughout the perioperative period. Laboratory findings of severe anemia and hypoalbuminemia indicated significant metabolic compromise, prompting preoperative correction with PN and iron supplementation. Differential diagnosis excluded pregnancy-related anemia as the sole cause of these abnormalities, confirming nutritional depletion secondary to malignancy and chronic inflammation.

PN was implemented as part of a stepwise therapeutic strategy to maintain adequate caloric and protein intake when enteral feeding was not feasible. During the early postoperative phase, PN supported metabolic stability and wound healing while protecting the anastomosis. As bowel function recovered-evidenced by the return of bowel sounds, passage of flatus, and tolerance of clear fluids-nutrition was transitioned to enteral and then oral feeding. Adjustments to therapy were based on biochemical improvement, gastrointestinal tolerance, and patient feedback. The patient adhered well to all recommendations, achieved progressive normalization of laboratory parameters, and experienced no nutritional complications. This integrated, evidence-based approach demonstrates how diagnostic assessment, individualized intervention, patient compliance, and continuous evaluation together optimize perioperative recovery.

While minimally invasive surgery is preferred, complex pregnancy-associated cases may require open combined procedures to ensure maternal-fetal safety. Preoperative anxiety can exacerbate stress responses, emphasizing the role of psychological support and close monitoring. Multidisciplinary collaboration-including obstetrics, surgery, ICU, nutrition, and psychological services-was critical to optimize outcome.

Pregnancy-associated colorectal cancer is rare, and its symptoms-abdominal distension, back pain, and anemia-often mimic physiological changes in late pregnancy, potentially delaying diagnosis. In comparison with previous reports, this case demonstrates unique, integrated perioperative nursing strategies, including individualized pain management with PCA, tailored nutritional support (PN and EN), proactive complication prevention, psychological support, and maternal–neonatal separation management. These interventions facilitated optimal maternal recovery and favorable postoperative outcomes. Clinically, the case underscores the importance of multidisciplinary collaboration, evidence-based nursing care, and personalized intervention plans for complex oncological–obstetric conditions. However, the study is limited by its single-case design and the age of the case, which may affect generalizability. Further research with larger cohorts is warranted to validate and expand these nursing strategies for broader clinical application.

## 4 Conclusions

In conclusion, integrated high-quality nursing for a late-pregnancy patient with colorectal cancer contributed to favorable postoperative outcomes. This case underscores the importance of tailored perioperative care and multidisciplinary collaboration in managing complex oncological-obstetrical conditions.

## Data Availability

The original contributions presented in the study are included in the article/supplementary material. Further inquiries can be directed to the corresponding author.
